# Sharing self-related information is associated with intrinsic functional connectivity of cortical midline brain regions

**DOI:** 10.1038/srep22491

**Published:** 2016-03-07

**Authors:** Dar Meshi, Loreen Mamerow, Evgeniya Kirilina, Carmen Morawetz, Daniel S. Margulies, Hauke R. Heekeren

**Affiliations:** 1 Department of Education and Psychology, Freie Universität Berlin, Habelschwerdter Allee 45, 14195 Berlin, Germany; 2 Center for Cognitive Neuroscience Berlin, Habelschwerdter Allee 45, 14195 Berlin, Germany; 3 Max Planck Research Group for Neuroanatomy & Connectivity, Max Planck Institute for Human Cognitive and Brain Sciences, Stephanstrasse 1a, 04103 Leipzig, Germany

## Abstract

Human beings are social animals and they vary in the degree to which they share information about themselves with others. Although brain networks involved in self-related cognition have been identified, especially via the use of resting-state experiments, the neural circuitry underlying individual differences in the sharing of self-related information is currently unknown. Therefore, we investigated the intrinsic functional organization of the brain with respect to participants’ degree of self-related information sharing using resting state functional magnetic resonance imaging and self-reported social media use. We conducted seed-based correlation analyses in cortical midline regions previously shown in meta-analyses to be involved in self-referential cognition: the medial prefrontal cortex (MPFC), central precuneus (CP), and caudal anterior cingulate cortex (CACC). We examined whether and how functional connectivity between these regions and the rest of the brain was associated with participants’ degree of self-related information sharing. Analyses revealed associations between the MPFC and right dorsolateral prefrontal cortex (DLPFC), as well as the CP with the right DLPFC, the left lateral orbitofrontal cortex and left anterior temporal pole. These findings extend our present knowledge of functional brain connectivity, specifically demonstrating how the brain’s intrinsic functional organization relates to individual differences in the sharing of self-related information.

Human beings like to share information about themselves. Around 30–40% of everyday speech contains personal information about the speaker^1^,^2^, and people will forgo money for the opportunity to disclose information about themselves^3^. Importantly, humans display differences in the degree to which they share self-relevant information^4^. These differences are crucial considering that the ability to present oneself effectively to others, which includes the sharing of self-related information, is one of the most vital skills in human social life^5^. In support of this, self-presentation plays an important role in occupational success, romantic attraction, making friends, and other desirable aspects of life^6^,^7^,^8^,^9^,^10^.

Social psychologists have proposed cognitive process models for the decision to disclose self-related information^11^,^12^,^13^,^14^. In general, these models require the self-disclosing person to keep self-related information in mind as they decide whether to share it. The person also considers the availability of a disclosure target. If a target is available, the self-disclosing person enters into a process where various positive and negative aspects of sharing the information with the target are weighed. To explain, a person may consider how sharing information about themselves will make them feel and what there is to gain or lose from sharing. They may also consider how their disclosure target may react to hearing this information. The person finally decides whether or not to share the self-relevant information. Therefore, this entire decision process concerning the sharing of self-related information involves various types of cognition, such as self-referential thought, social cognition (e.g., mentalizing), reward-related processing, and executive functioning (e.g., working memory).

In contrast to the above-mentioned comprehensive theoretical concepts and behavioral findings, research into the neurocognitive processes related to sharing self-related information has been limited. Thus far, only one study has been performed^3^. The authors used functional magnetic resonance imaging (fMRI) to find that both the medial prefrontal cortex (MPFC) and nucleus accumbens were activated when people decided to share self-related information. Other research has simply looked at self-referential cognition. A meta-analysis of event-related fMRI experiments revealed clusters of activation at three cortical midline regions when thinking about oneself: the MPFC, the central precuneus (CP), and the caudal anterior cingulate cortex (CACC)^15^. Resting-state fMRI has also helped reveal the MPFC and CP to be involved in self-referential cognition, when examined in conjunction with a task^16^,^17^. Other resting-state studies have demonstrated that variance in intrinsic functional connectivity reflects individual differences in traits and behaviors^18^,^19^,^20^,^21^. These studies provided important insights into the neural foundations of personality traits and their associated behaviors. Importantly however, individual differences in intrinsic connectivity have not yet been investigated with respect to the sharing of self-related information. Furthermore, the examination of individual differences in personality traits and brain measures has mostly neglected traits related to social media use (for a review see Meshi *et al.*^22^).

Therefore, to examine individual differences in the network underlying self-related information sharing, we collected resting-state fMRI and conducted exploratory analyses focused on three key cortical midline regions, mentioned above, which had previously been associated with self-referential cognition, the MPFC, CP and CACC^15^. We hypothesized that the intrinsic functional connectivity of these three regions to other brain regions involved in the self-disclosure process would be associated with the degree to which individuals share self-related information. To explain, a major aspect of the above-described self-disclosure cognitive process model is self-cognition, via keeping in mind self-related information. There should be a direct relation between the amount of self-related cognition and the amount of self-related information sharing. This should require processing in the MPFC, CP and CACC, as well as communication to other brain regions involved in the self-disclosure process. The self-disclosure cognitive process model also involves social cognition, reward-related processing, and executive functioning. Social cognition, such as mentalizing, engages both the MPFC and CP, as well as the temporoparietal junction and temporal poles, as demonstrated by recent meta-analyses^23^,^24^; reward-related processing regarding the sharing of self-related information involves the MPFC and the ventral striatum^3^; and executive functioning, such as working memory, engages the dorsolateral prefrontal cortex (DLPFC), ACC, and other prefrontal regions, also demonstrated by recent meta-analyses^25^,^26^. These regions are therefore candidates for demonstrating individual differences in intrinsic functional connectivity with cortical midline regions with reference to the amount of self-related sharing of an individual.

To address our research question, we decided to capitalize on self-related sharing on social media. People can share self-related information in various ways, for example by talking face-to-face, conversing on the phone and writing letters. With the advent of social networking websites, such as Facebook and Twitter, self-related information can now be easily broadcast over the Internet. People can share information about themselves with friends and others, potentially to a large number of people. A great deal of the shared information on social networking sites is directly related to the person sharing the information^27^. Therefore, we employed the sharing of self-related information on social media, specifically the website, Facebook, as a proxy for the sharing of self-related information in the real world. This was accomplished by using an established questionnaire concerning self-related sharing behavior on Facebook^28^.

## Methods

### Participants

We recruited 35 healthy, right-handed participants (14 male) between 19 and 34 years of age (mean = 25.7, s.d. = 3.7). All participants had no history of psychiatric disorder and gave written informed consent. All experimental protocols were approved by the Freie Universität Berlin ethics committee, and all data collection and analyses were carried out in accordance with the approved guidelines. All participants had personal profiles on the Facebook website at the time of the study. Participants had been using Facebook for an average of 57.6 months (s.d. = 14.7; range = 31–107 months), and had an average of 312.8 friends (s.d. = 212.2; range = 35–864 friends).

On the day of testing, participants were placed in the scanner and appropriate imaging data were acquired. After scanning, all participants completed the questionnaires described below. To note, we have reported all measures and conditions, and there were no data exclusions. In addition, the number of participants was determined before data collection in accordance with best-practices in neuroimaging research^29^ and to provide sufficient power to detect significant effects^30^,^31^.

### Self-related sharing assessment

Participants were given a survey to assess the degree that they share self-related information, both written and visual, on Facebook^28^. The author of this survey, Carpenter, established the survey’s construct validity and internal consistency. To note, Carpenter termed the construct “self-promotion”, but self-deprecating information can also be shared on Facebook, and this behavior is included in the survey, which does not ask for the valence of the shared information (see survey questions below). Therefore, we avoid the assumption that all self-related information shared on Facebook is self-promoting, and we term the survey construct, “self-related sharing”. We also added one question (#2 below) to directly ask about the sharing of self-related website links, a self-sharing behavior on Facebook that was previously not addressed. Addition of this question did not affect the internal consistency of the survey; analysis of our 35 participants’ responses revealed a Cronbach’s α of 0.721 indicating that our questionnaire had good reliability. The survey questions were as follows:

1. How frequently do you update your profile information (for example: “about me”) on Facebook?
2. How frequently do you post links related to yourself or your activities to your Facebook timeline/wall?
3. How frequently do you update your status on Facebook?
4. How frequently do you change your profile picture/cover photo on Facebook?
5. How frequently do you post pictures of yourself or your activities on Facebook?
6. How frequently do you tag yourself in pictures/videos on Facebook?

Participants responded on a 5-point Likert scale (1 = Never to 5 = All the time) and responses for all six items were added to obtain a total score. This total score is referred to as the “self-related sharing score” throughout this manuscript. Participants demonstrated a broad range of self-related sharing scores (range = 6–20, mean = 11.8, standard deviation = 2.9). To note, self-related sharing did not correlate with number of Facebook friends (Pearson’s r = 0.258, p = 0.135). In addition, participants filled out the Facebook Intensity Scale^32^, although this data was not used in any analysis as it is not directly related to our question of interest.

### Personality surveys

In addition to the self-related sharing assessment, participants also filled out the following questionnaires: Rosenberg Self Esteem Scale^33^; Reynolds Social Desirability Scale (form C)^34^; Narcissistic Personality Inventory (16 question)^35^; and the Mehrabian Conformity Scale^36^. These measures were used to further elucidate factors driving self-related sharing.

### Imaging procedure

Scanning was performed at the Center for Cognitive Neuroscience Berlin at the Freie Universität Berlin, Germany using a 3T Siemens Trio scanner (Siemens Healthcare Diagnostics GmbH, Erlangen, Germany) equipped with a 12-channel receiver MR-coil. While in the scanner, participants saw a low-luminosity fixation cross on LCD-goggles (Resonance Technology Inc., Northridge, California). First, fieldmaps were acquired using a dual echo 2D gradient echo sequence with echo times (TE) of 4.92 and 7.38 ms, and a repetition time (TR) of 488 ms. Next, whole-brain resting-state functional images were acquired with a echo-planar (EP) T2*-weighted gradient echo sequence (TE = 30 ms, TR = 2.3 s, matrix = 64 × 64, flip angle =90°, in-plane resolution 3 × 3 mm^2^, interslice gap =0.3 mm). A total of 37 oblique-axial slices (3 mm slice thickness) parallel to the anterior commissure-posterior commissure line were collected per volume in an interleaved order. A total of 200 volumes were collected per run, resulting in a run time of 7 min and 40 s. Two runs were collected sequentially in the same session for each participant. After resting-state data acquisition, anatomical images were acquired using a T1-weighted MPRage protocol (176 sagittal slices, isotropic resolution 1 × 1 × 1 mm^3^, 256 × 256 data acquisition matrix).

### Resting state fMRI data preprocessing

Neuroimaging data were analyzed with SPM8 (Statistical Parametric Mapping, Wellcome Institute for Cognitive Neurology, London, UK; RRID: nif-0000-00343) and the data processing assistant for resting-state fMRI (DPARSF; RRID: nlx_155735)^37^. The first 10 volumes of each functional run were removed and the remaining 190 volumes were corrected for differences in slice acquisition time. Fieldmaps were then utilized to correct the functional data for susceptibility-related distortions, and the data were motion-corrected using a least squares approach and a six-parameter (rigid body) linear transformation. Data were then spatially normalized and additional regression of nuisance covariates was applied, during which the functional data were detrended and corrected for the six head movement parameters (X, Y, Z, pitch, yaw, and roll), global signal, white matter (WM) signal, and cerebrospinal fluid (CSF) signal^37^. This step controls for physiological noise, such as fluctuations related to motion, as well as cardiac and respiratory cycles^38^. Data were then band pass filtered (0.01–0.08 Hz) to eliminate low frequency fluctuations, and smoothed with a 6-mm full-width-at-half-maximum Gaussian kernel.

To note, we performed a global signal regression during the preprocessing of our neuroimaging data to reduce the effect of physiological noise^39^. Some studies have suggested that global signal regression may also introduce artifactual anticorrelations (e.g., Murphy *et al.* 2009)^40^. Recent results, however, have suggested that false correlations are suppressed, and connection specificity is improved by global signal regression, and furthermore, anticorrelations have been observed even without global signal regression^38^,^41^,^42^. Nevertheless, after our primary analysis (see Results), we set out to establish that our findings were independent of the preprocessing method; in other words, to demonstrate that our findings were not artifacts of the global signal regression performed in preprocessing and to qualitatively confirm the spatial locations of our findings. To do this, we performed a separate analysis via alternate preprocessing; regression of the global signal, WM signal, and CSF signal was replaced with a set of 10 auxiliary physiological regressors generated using the CompCor method^43^. Briefly, we first generated CSF and WM masks based on the segmented T1-weighted images for each participant. These masks were smoothed with a Gaussian kernel of 8 mm full-width-at-half-maximum and cut at a threshold of 0.95 to provide more conservative masks. Time courses of the first five principal components extracted from the EP images masked with the CSF mask, and the first five principle components extracted from the EP images masked with the WM mask, were used as 10 auxiliary regressors. To note, the EP images used for this principal component analysis had not undergone smoothing and normalization.

### Seed generation

Our research goal was to investigate resting-state functional connectivity related to self-related information sharing. A prerequisite of self-related information sharing is self-referential cognition. Therefore, we focused our analyses on brain regions which were previously shown to be involved in self-referential cognition. Northoff and colleagues conducted a meta-analysis of 27 neuroimaging studies which compared the neural processing of stimuli related to the self and non-self-related stimuli^15^. The authors’ primary factor analysis revealed 3 medially located clusters in the brain.

**Figure 1:**
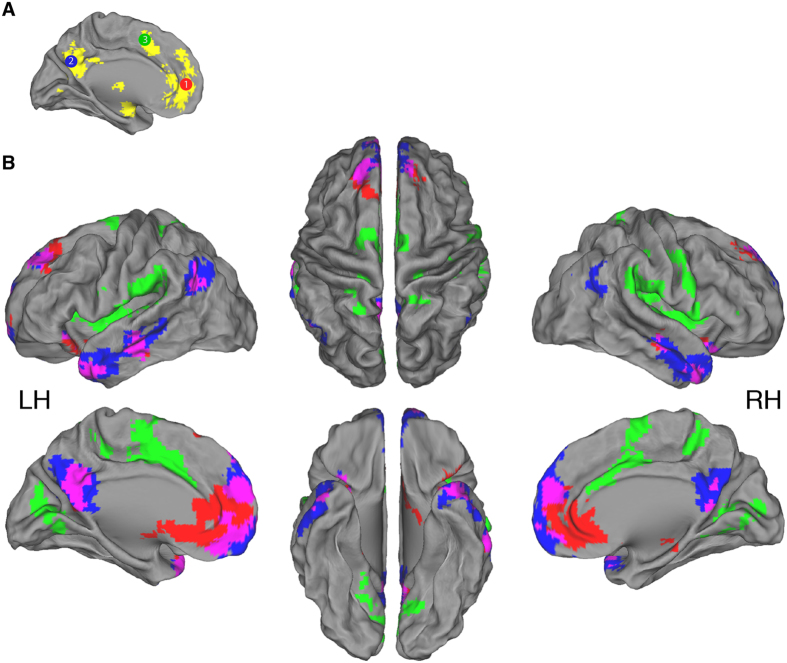
Intrinsic functional connectivity of three seeds in brain regions previously demonstrated to play a role in self-referential cognition. (**A**) Representation of location of the three seeds (10 mm diameter) with identifying color and number: 1. MPFC = red, 2. CP = blue, 3. CACC = green. Seed location is based on a meta-analysis of self-referential cognition^15^. For confirmation that these regions are important for self-cognition, we queried the Neurosynth database with the search term “self” (http://neurosynth.org)^44^. This resulted in the yellow activation map (thresholded at z-value >4.0). Substantial overlap between the seed regions and the Neurosynth map is apparent. (**B**) Brain surface maps illustrating regions with significant intrinsic functional connectivity with each of the three seed regions. Areas showing connectivity to Seeds 1, 2 and 3 are shown in red, blue and green, respectively. Overlap between Seed 1 and Seed 2 is shown in purple. All results are whole-brain FWE cluster-level corrected at p < 0.05 after setting the voxel-level uncorrected threshold to p < 0.001. *n* = 35; LH = Left hemisphere; RH = Right hemisphere.

To further confirm that these cortical midline regions are relevant to the self, we queried the Neurosynth database with the search term “self” (http://neurosynth.org)^44^. The Neurosynth database at the time of query consisted of 5,809 neuroimaging studies, of which 206 studies were positive for the search term and therefore included in an automated meta-analysis. We downloaded the resulting forward-inference statistical map, which was thresholded at z-value >4.0 (Fig. 1A, yellow). Spatial comparison of this map and the above-described seeds generated from the Northoff *et al.* meta-analysis reveals a high overlap.

Therefore, we created spherical seeds, 10 mm in diameter, located at the center of each of the 3 clusters resulting from Northoff *et al.*’s analysis (Fig. 1A): Seed 1 in the MPFC (MNI coordinates: −2, 49, 7), Seed 2 in the CP (−3, −61, 31), and Seed 3 in the CACC (−1, 10, 49). To note, each seed extended into the right hemisphere due to their 5 mm radius.

We also investigated ventral striatum connectivity with respect to self-related sharing, due to the finding that sharing self-related information involves the ventral striatum^3^. We created spherical seeds, 10 mm in diameter, located bilaterally in the ventral striatum based on prior functional connectivity analysis of this area^45^ (Right: 9, 9, −8; Left: −9, 9, −8). To note, the analyses described below did not reveal any significant clusters that survived correction for multiple comparisons (see threshold below).

### Individual-level analysis

The average time-series of each seed, in each participant, was extracted and a correlation was calculated with each voxel. Correlation (*r*-value) maps from both resting-state runs were then converted into *z*-values by a Fisher *r*-to-*z* transform to improve the normality of the scores. The two z-value maps for each run were then averaged into one map. Thus, all voxels in the resulting standardized *z*-value maps demonstrated the degree of correlation with the corresponding seed for each participant.

### Group-level analysis

**Figure 2:**
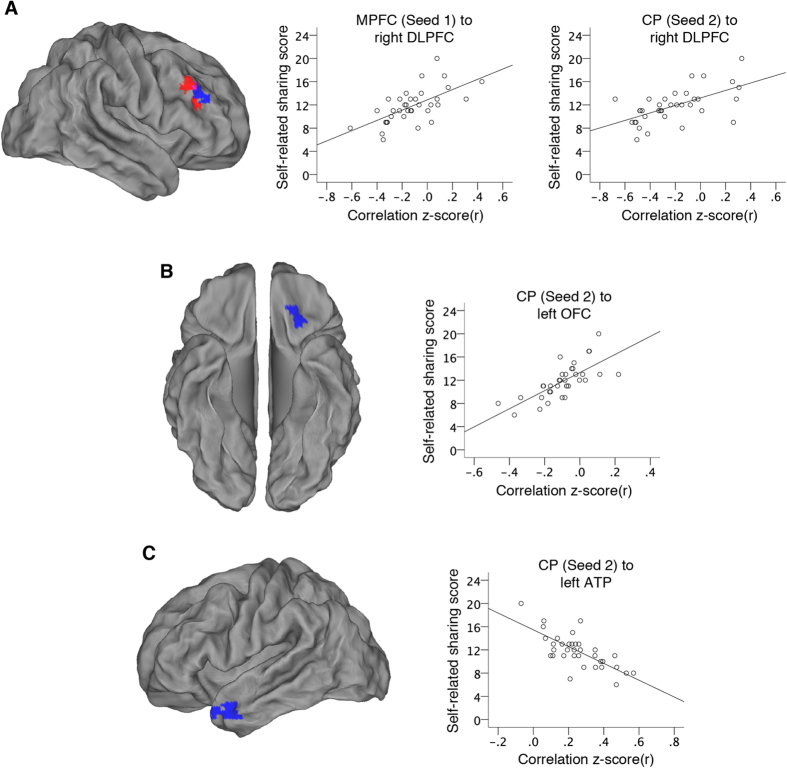
Intrinsic functional connectivity of cortical midline regions previously demonstrated to play a role in self-referential cognition is associated with self-related information sharing on social media across participants. (**A**) Connectivity of the MPFC and the CP to the right DLPFC is positively associated with self-related sharing score across participants (connectivity to Seed 1 in the MPFC = red, Seed 2 in the CP = blue). Overlap in the right DLPFC between results from MPFC and CP is 22 voxels (purple). Plots depict participants’ resting-state activation correlations between either the MPFC or CP and the right DLPFC with respect to their self-related sharing scores. (**B**) Connectivity of the CP to the left lateral OFC is positively associated with self-related sharing score across participants. Plot depicts participants’ resting-state activation correlations between the CP and the left lateral OFC with respect to their self-related sharing scores. (**C**) Connectivity of the CP to the left ATP is negatively associated with self-related sharing score across participants. Plot depicts participants’ resting-state activation correlations between the CP and the left ATP with respect to their self-related sharing scores. All results are whole-brain FWE cluster-level corrected at p < 0.05 after setting the voxel-level uncorrected threshold to p < 0.001. All scatter plots are solely for illustrative purposes (e.g., to show the absence of outliers), and are not used for statistical inference.

For each seed, individual *z*-value maps were analyzed with a random-effect one-sample t-test to identify voxels with a significant correlation with the seed time-series at the group level. In the same model, a regressor containing each participant’s self-related sharing score was entered as a covariate of interest. This regressor was orthogonalized with respect to the main effect group regressor to capture only linear parametric variance in the imaging data specifically due to self-related sharing. Significance was set with a whole-brain family-wise error (FWE) cluster-level corrected threshold of p < 0.05 after setting the voxel-level uncorrected threshold to p < 0.001. This analysis resulted in two types of *z*-statistic maps: 1. Maps of voxels exhibiting significant intrinsic functional connectivity with the seed across all participants (Fig. 1), and 2. Maps of voxels whose functional connectivity with the seed correlated with each participant’s individual self-related sharing score (Fig. 2).

To note, our exact approach of examining resting state functional connectivity between seed regions and the whole brain, while maintaining hypotheses regarding selected areas, has been previously implemented in many other individual-differences studies to date (for a review see Vaidya & Gordon^46^).

## Results

### Personality construct assessment

We set out to examine intrinsic functional connectivity with respect to the degree to which individuals share self-related information. Before performing the analysis with resting-state fMRI data, we were concerned that more general personality constructs could account for the variance we would observe with respect to self-related sharing. Therefore, participants also filled out questionnaires for social desirability, self-esteem, narcissism and conformity, because we deemed these personality traits as likely to affect self-related sharing. We estimated a linear regression, with scores from these personality surveys as independent variables and self-related sharing scores as the dependent variable. The model was not significant; neither single predictors nor the model as a whole was significant (Adjusted R^2^ = −0.03, F_(4,30)_ = 0.749, p = 0.566; standardized betas: social desirability = −0.035; self-esteem =0.013; narcissism =0.306; conformity =0.058; all p’s >0.1). Therefore, self-related sharing is not accounted for by these other personality traits, and they were not included as covariates in the resting-state fMRI analysis.

### Intrinsic functional connectivity

The results of the random-effect group analysis of resting-state connectivity are presented in Fig. 1B and Table 1. Analysis with Seed 1, located in the MPFC, revealed intrinsic functional connectivity to the precuneus and the left anterior temporal pole (ATP). Significant connectivity to the middle temporal gyrus in both the left and right hemispheres was also observed. Analysis with Seed 2, located in the CP, revealed intrinsic functional connectivity to the MPFC, as well as the ATP’s of both the left and right hemispheres. Analysis with Seed 3, located in the CACC, revealed significant connectivity to the insular cortex in both the left and right hemispheres, as well as the precuneus.

### Self-related sharing and intrinsic functional connectivity

We conducted exploratory analyses of the resting-state fMRI data with self-related sharing score as a covariate (Table 2). Analysis with Seed 1 revealed that intrinsic functional connectivity between the MPFC and the right DLPFC, specifically within the middle frontal gyrus, is positively associated with self-related sharing score across participants (Fig. 2A). Analysis with Seed 2 revealed that connectivity between the CP and the right DLPFC is also positively associated with self-related sharing score across participants (Fig. 2A). The results in the right DLPFC from Seed 1 and Seed 2 overlap to an extent of 22 voxels. Analysis with Seed 2 also revealed that connectivity between the CP and the left lateral OFC is positively associated with self-related sharing score across participants (Fig. 2B), while connectivity between the CP and the left ATP is negatively associated with self-related sharing score across participants (Fig. 2C). To note, all but one of these correlations in the left ATP was positive (Fig. 2C plot). Analysis with Seed 3 revealed no significant associations with self-related sharing.

### Analysis with alternative preprocessing

We performed an alternative analysis with the CompCor preprocessing method to establish that our findings were independent of the preprocessing method (Table 3). We set significance at the voxel-level uncorrected threshold of p < 0.001 with a minimum cluster size of 10 voxels. Analysis with Seed 1 qualitatively corroborates our previous results; connectivity between the MPFC and the right DLPFC is positively associated with self-related sharing score across participants (peak MNI coordinates: 45, 24, 36; cluster size: 11 voxels). Analysis with Seed 2 revealed connectivity to the left lateral OFC (−21, 33, −15; 10 voxels) and right paracingulate gyrus (6, 36, 3; 11 voxels) that is positively associated with self-related sharing score across participants, and connectivity to the left ATP that is negatively associated with self-related sharing score (−48, 9, −21; 20 voxels). Unlike our previous analysis, our secondary, alternative analysis with Seed 3 in the CACC revealed significant associations; connectivity between the CACC and both the right amygdala (21, 0, −15; 17 voxels) and left lateral OFC (−21, 30, −18; 15 voxels) was negatively associated with self-related sharing score across participants.

To note, when we reduced the threshold of significance to p < 0.005, we also observed a positive association between self-related sharing score and connectivity of Seed 2 in the CP to the right DLPFC (42, 33, 33; 64 voxels). Therefore, the results from our primary analysis are present and overlap with clusters resulting from our secondary, alternative analysis. This confirms the spatial location of our findings. It does appear that the CompCor method affected the effect size of the results, because a lower threshold of significance was used. Although, important to note, all results remained when using a recommended threshold (p < 0.005; cluster size ≥10) to minimize Type I and II error rates^47^, or a more conservative threshold (p < 0.001; cluster size ≥10). The less significant findings could be due to more residual noise after the CompCor method as compared to global signal regression.

## Discussion

In the present study, we examined whether and how individual differences in the sharing of self-related information on social media are associated with intrinsic functional brain organization. To assess the sharing of self-related information, we used self-report of social media sharing and computed a self-related sharing score for each participant. We focused our analyses on brain regions demonstrated by a meta-analysis to be involved in self-referential cognition^15^. Thus, we analyzed the resting-state data by placing seeds in these self-referential cortical midline areas: the MPFC, CP, and CACC. To confirm that our resting-state analyses were being performed correctly, we first examined the intrinsic functional connectivity of these three seed regions without respect to self-related sharing score. Our results were in line with previous resting-state research with seeds in these brain regions^48^,^49^. We next interrogated the data with respect to participants’ self-related sharing score. We found that, across participants, connectivity of both the MPFC and CP, but not connectivity of the CACC, to other brain regions is associated with the degree of self-related sharing.

In the context of the general cognitive model of self-disclosure presented in the introduction, our data can be interpreted as follows. As mentioned previously, the self-disclosing person needs to keep self-related information in mind. This is why we chose to investigate three brain regions already demonstrated to be involved in self-referential cognition: the MPFC, CP and CACC^15^. We found that the connectivity of both the MPFC and CP, but not connectivity of the CACC, to other brain regions is associated with the degree of self-related sharing across participants. Our results may be due to the MPFC and posteromedial cortex, which contains the CP, being core hubs of self-referential processing in the default-mode network^16^,^50^. The CACC is not considered a core hub of self-referential processing in the resting state. Furthermore, the MPFC and CP, not the CACC, have previously been demonstrated to be involved in the sharing of self-related information^3^. This paper by Tamir and Mitchell contains several neuroimaging studies on self-disclosure, with most showing MPFC activations, and a couple showing activations in the precuneus. Thus, these regions which were previously demonstrated, in a task-related manner, to process the act of sharing self-related information, overlap to an extent with the regions we found to demonstrate intrinsic functional connectivity related to participants’ self-related sharing score, the MPFC and CP.

Keeping self-related information in mind during the decision to share should also require communication between brain regions involved in self-referential cognition and regions involved in working memory. Meta-analyses of event-related fMRI studies demonstrate that the DLPFC plays a role in general executive functioning^25^, and more specifically working memory^26^. Furthermore, intrinsic functional connectivity of the right DLPFC is related to working memory^19^. We found that the greater the intrinsic connectivity between the right DLPFC and both the MPFC and CP, the more likely one is to share self-related information on social media. Although our results demonstrate there is an involvement of DLPFC intrinsic connectivity in the sharing of self-related information, we cannot determine which aspect of our cognitive process model this connectivity finding represents. Nevertheless, we speculate that people who share more about themselves may have intrinsic brain organization that allows them to better keep self-related information in working memory, or alternatively, they may be simply keeping more self-related information in mind when left to their own thoughts.

Furthermore, as mentioned above, there is a social component to sharing self-related information; a person has to think about whom to share with and how that target will react. This should require communication between brain regions involved in self-referential cognition and regions involved in social cognition. Both the OFC and the ATP play a role in social cognitive processes. OFC lesion studies firmly establish its role in social cognition; “No prefrontal lesion is likely to have as great an impact on social behavior as the orbitofrontal lesion”^51^. Social behaviors have also been related to lateral OFC gray matter density and functional processing^52^,^53^,^54^,^55^. We found that the greater the intrinsic connectivity between the CP and the left lateral OFC, the more likely one is to share self-related information on social media. Interestingly, with our secondary analysis, we found that the less the connectivity between the CACC and the left lateral OFC, the more likely one is to share self-related information on social media. This result further implicates connectivity of the left lateral OFC in the sharing of self-related information. With regards to the ATP, a meta-analysis of event-related fMRI studies demonstrated its involvement when thinking about the mental states of others^24^, and ATP gray matter density correlates with social behavior as well^56^. We found that the greater the intrinsic connectivity between the CP and the left ATP, the less likely one is to share self-related information on social media. To note, this region of the left ATP was positively connected to both the MPFC and the CP (Fig. 1B). It appears that our findings about people’s intrinsic brain organization support the idea put forth in cognitive models of self-disclosure, that people need to think about others and how they will react when sharing self-related information^11^,^12^,^13^.

With our personality construct regression analysis, we demonstrated that individual differences in self-related sharing on social media cannot be accounted for by differences in social desirability, self-esteem, narcissism or conformity. Important to note, our self-related sharing construct was previously shown to be associated with sub-scale aspects of narcissism^28^. This discrepancy could be due to our using a different narcissism scale^35^. In addition, other more general measures of Facebook use have been linked with self-esteem and narcissism^57^,^58^,^59^. Finally, the personality surveys we selected for inclusion in this experiment are by no means exhaustive. Future research may yield a relationship between self-related sharing and extroversion or empathy, for example.

We employed the sharing of self-related information on social media as a proxy for the sharing of self-related information in the real world. This surrogate approach has been used before in neuroimaging research; two studies used number of Facebook friends as a proxy for social network size^53^,^60^, and another other study used intensity of Facebook use as a proxy for a real-world behavior aimed at reputation management^59^. To note, the real-world behavior of an individual can vary from that individual’s behavior on social media. For example, people disclose more self-related information in computer mediated communication, as compared to face-to-face communication^61^. This should be kept in mind when interpreting our results. However, with a significant portion of the world’s population regularly using social networking websites, over 1.5 billion people on Facebook alone^62^, research into understanding the sharing of self-related information, especially on social networking sites, is warranted^22^.

Taken together, our findings demonstrate that several brain regions, the right DLPFC, left lateral OFC and left ATP, which have been previously demonstrated to function in either working memory or social cognition, respectively, are actively linked with previously established regions involved in self-referential cognition, the MPFC and CP, during the resting state. We showed that the coupling between the MPFC and CP and the three previously mentioned brain regions is associated with the amount that people share about themselves with others, specifically on social media. Although we performed a resting-state experiment without a task, our findings suggest that the act of sharing self-related information involves connectivity between these regions. The implications of this are broad and lead to several questions. First, what is the causal nature of the relationship between brain connectivity and self-disclosure? For example, is the connectivity established at a young age, driving the behavior, or does repeated sharing of self-related information alter the intrinsic functional connectivity of the brain between these specific regions? Second, which brain areas are required for the act of self-related information sharing? Future experiments will be able to target the regions we revealed. For example, does creating a temporary lesion in the right DLPFC with repeated transcranial magnetic stimulation decrease self-related information sharing? Conversely, does a temporary lesion of the left ATP increase this behavior? Next, to what degree is there overlap between the neural substrates involved in self-related information sharing and non-self-related information sharing? Different regions may be involved for these different behaviors, or different computations may take place in the same brain regions. Finally, how and to what extent are self-related information sharing and social cognition related to each other? Mentalizing is an integral part of self-disclosure, and individuals may mentalize to different degrees when sharing self-related information. Future resting-state research can examine the intrinsic functional connectivity of brain regions involved in social cognition^23^ with respect to both the degree of mentalizing and self-related information sharing. To note, recent evidence exists that the social brain network mapped by Schilbach and colleagues^23^ is found to be altered in pathological states^63^, which is important because the sharing of self-related information, and participation in social networks, can influence personal wellbeing and quality of life^64^,^65^,^66^.

In conclusion, sharing self-related information is a ubiquitous behavior that may provide adaptive advantages for individuals^67^,^68^. Effectively presenting oneself to others, which includes the sharing of self-related information, is a crucially important social skill^5^, playing a role in occupational success, romantic attraction, making friends, and other desirable aspects of life^6^,^7^,^8^,^9^,^10^. Our findings shed light on the neural systems supporting this self-disclosure, providing an important step towards understanding the human social behavior of sharing information about the self.

## Additional Information

**How to cite this article**: Meshi, D. *et al.* Sharing self-related information is associated with intrinsic functional connectivity of cortical midline brain regions. *Sci. Rep.* **6**, 22491; doi: 10.1038/srep22491 (2016).

## Figures and Tables

**Table 1:** Brain regions demonstrating significant positive intrinsic functional connectivity with seeds in cortical midline areas.

Region	MNI Coordinates			Cluster size	Peak z
	x	y	z		
Seed 1 in MPFC					
L/R Precuneus	−6	−57	21	226	4.60
L Anterior temporal pole	−42	18	−27	203	4.17
L Middle temporal gyrus	−60	−21	−12	144	4.84
R Insular cortex	39	15	−15	138	4.62
L/R Brain stem	3	−21	−18	81	4.75
L Angular gyrus	−42	−54	24	79	3.66
R Middle temporal gyrus	69	−9	−15	71	4.80
Seed 2 in CP					
L/R Medial prefrontal cortex	0	57	21	1159	5.32
L Anterior temporal pole	−48	6	−24	700	6.00
R Anterior temporal pole	42	18	−27	524	5.16
L Angular gyrus	−45	−60	30	356	5.45
R Cerebellum	30	−81	−30	202	5.03
R Angular gyrus	60	−54	42	181	4.35
L Cerebellum	−27	−75	−33	158	4.97
Seed 3 in CACC					
L Insular cortex	−36	−3	−3	1094	5.86
R Insular cortex	36	12	9	731	5.23
L Occipital cortex	−15	−69	3	579	5.47
R Central opercular cortex	57	−12	15	519	4.70
R Precuneus	12	−42	54	131	4.92
L Precentral gyrus	−12	−33	51	106	4.61
L Postcentral gyrus	−12	−48	60	59	4.16

Whole-brain FWE cluster-level corrected at p < 0.05 after setting the voxel-level uncorrected threshold to p < 0.001. L = Left; R = Right.

**Table 2:**
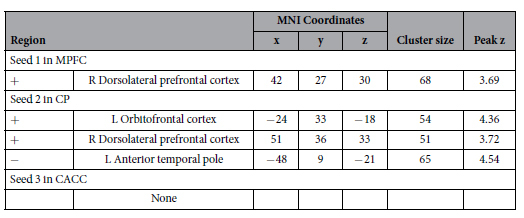
Brain regions associated with self-related sharing across participants.

Whole-brain FWE cluster-level corrected at p < 0.05 after setting the voxel-level uncorrected threshold to p < 0.001. +/− =Type of association.

**Table 3:**
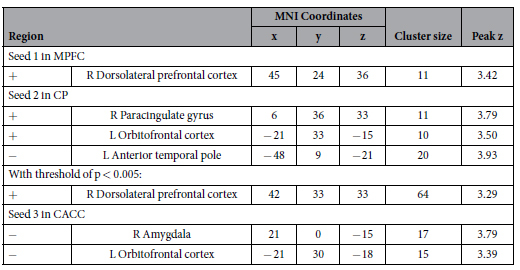
CompCor analysis confirming brain regions associated with self-related sharing across participants.

Whole-brain analysis at a voxel-level uncorrected threshold of p < 0.001 with a minimum cluster size of 10 voxels. +/− =Type of association.
